# Hydroxypropyl-β-Cyclodextrin-Complexed Resveratrol Enhanced Antitumor Activity in a Cervical Cancer Model: *In Vivo* Analysis

**DOI:** 10.3389/fphar.2021.573909

**Published:** 2021-04-15

**Authors:** Xincai Hao, Xiaodong Sun, Haizhen Zhu, Lixia Xie, Xuanbin Wang, Nan Jiang, Pan Fu, Ming Sang

**Affiliations:** ^1^ Hubei Clinical Institute of Parkinson’s Disease at Xiangyang No .1 People’s Hospital, Hubei Key Laboratory of Wudang Local Chinese Medicine Research, Hubei University of Medicine, Shiyan, China; ^2^ Hubei Province Hospital of Traditional Chinese Medicine, Hubei Province Academy of Traditional Chinese Medicine, Wuhan, China

**Keywords:** hydroxypropyl-β-cyclodextrin, resveratrol, cervical cancer, HPV E6 and E7, in vivo

## Abstract

*Trans*-resveratrol (RES) exhibits a wide range of biological activities. Various methodological approaches have been established to improve the pharmacokinetic properties of RES. Moreover, additional *in vivo* studies are required to support clinical application. In this study, RES/HP-β-CD (RHSD) inclusion complex was prepared and characterized by FTIR, PXRD, DSC and NMR data. The effect and potential mechanism of RHSD against cervical cancer were investigated in a mouse xenograft tumor model by qPCR assay, Western blot assay, and immunohistochemical assay. Results showed that RHSD significantly decreased tumor growth compared with free RES, while the effect of preventing tumor growth was more prominent *in vivo*. Notably, RHSD could inhibit tumor development by suppressing the expression of HPV E6 and E7 oncogenes and upregulating P53 and Rb1 protein in cervical cancer. These findings demonstrated that RHSD was safe and potential for development of a new oral administration drug to treat cervical cancer.

## Introduction

Cervical cancer is the second most common cancer of global cancer deaths among women (Ferlay et al., 2015). Over 500,000 new cases of cervical cancer are reported every year, and most cases are from developing countries (Chatterjee et al., 2019). HPV 16 and 18 are estimated to be associated with cervical cancer and precancerous lesions worldwide (Nubia et al., 2004; He et al., 2014). HPV E6 and E7 are two major oncoproteins encoded by HPV 16 and 18 (Tan et al., 2012), which play a central role in the onset of aging in HPV-positive cancer cells. High-risk HPV E6 and E7 proteins have been shown to promote tumorigenesis by promoting cell immortality and migration, changing cell cycle and apoptosis control, and avoiding host immune surveillance (Thomas and Chiang, 2005; Liu et al., 2009; De Sanjose et al., 2010; Ghittoni et al., 2010; Au Yeung et al., 2011). This cellular response is related to the regeneration of P53 and Rb1 anti-proliferative proteins (Scheffner et al., 1990; Dyson et al., 1992; Heck et al., 1992; Huibregtse et al., 1993; Taghizadeh et al., 2019). Therefore, inhibition of the E6/P53 and E7/Rb pathways is an effective therapeutic strategy for cervical cancer (Münger and Howley, 2002; He et al., 2014). Unfortunately, the current cancer prevention and treatment methods in developing countries have failed to significantly reduce cervical cancer mortality, because of the high costs originating from side effects of treatment. Therefore, non-toxic and low-cost natural products with anti-cancer properties have continued to attract great interest.

*Trans*-Resveratrol (3,5,4′-trihydroxy-trans-stilbene; RES), which belongs to the family of stilbestrol, is abundant in red wine, fruit, and vegetables. RES has shown a wide range of biological activities, including anti-inflammatory activity, antioxidant activity (Gambuti et al., 2004), cardiac protective action (Adrian et al., 2000), anti-aging (Hsu et al., 2014), and anticancer activity (Kulacki and Lamberti, 2008). Kim et al. have found that RES can suppress the migration and invasion of human cervical cancer cells (Kim et al., 2012). Liu et al. report that RES induce cervical cancer HeLa cell apoptosis through the activation and nuclear translocation promotion of FOXO3a (Liu et al., 2020). Li et al. have shown RES suppresses human cervical carcinoma cell proliferation and elevates apoptosis via the mitochondrial and p53 signaling pathways (Li et al., 2018). Chatterjee et al. and our team research also find that the anti-cervical cancer activities of RES are connected with expression of HPV E6 and E7 genes (Chatterjee et al., 2019; Sun et al., 2021). In addition, Clinical trials in humans have shown that RES is nontoxic to normal cells and are well-tolerated (Riche et al., 2013). However, the bioavailability of RES is not optimal due to its poor water solubility, which decreases its pharmacological effects because of its low absorption in oral administration.

Cyclodextrin (CD) inclusion complexes has been proved to be the most effective method to improve the solubility and dissolution of insoluble drugs (Tang et al., 2016). CD are equipped with a hydrophobic cavity to encapsulate another molecule forming a water-soluble complex through host–guest interactions. In particular, 2-Hydroxypropyl-β-CD (HP-β-CD), modified cyclodextrin derived from β-CD, has high medicinal values in terms of increasing the guest’s stability, oxidation and dehydration (Liu et al., 2006), improving dissolution rate, bioavailability (Loftsson and Brewster, 1996; Hsu et al., 2019), enhancing the permeation, prolonging the effect of medicine, reducing irritant and drug toxicity (Scavone et al., 2016; Tang et al., 2016). Previous research reported that the encapsulation of RES by HP-β-CD can increase its light stability, dissolution rate, and biological activity *in vitro* (Lucas-Abellán et al., 2008; Wei et al., 2017). However, *in vivo* studies are required to support its clinical application. To the best of our knowledge, no study on the RES/HP-β-CD (RHSD) inclusion complex against cervical cancer has been conducted. Therefore, the aim of this study was to investigate the anti-cervical cancer effects of RHSD *in vivo* and determine its related mechanism.

In this study, RHSD was prepared with the co-evaporation method under a 1:1 ration and characterized by Fourier transform infrared spectroscopy (FT-IR), powder X-ray diffraction (PXRD), and differential scanning calorimetry (DSC) and nuclear magnetic resonance (NMR). The solubility and dissolution rate were investigated *in vitro*, and the effect of the RSHD complex on cervical cancer was evaluated in a murine model of HeLa cell-induced cancer *in vivo*. Their preventive potential effect on cervical cancer was also evaluated.

## Materials and Methods

### Materials

Analytical grade RES (purity 98.0%) was purchased from Aladdin (Shanghai, China). HP-β-CD (average Mw = 1540 Da with 1.0 M substitution, hydroxypropyl moiety) were obtained from Aldrich (St. Louis, MO, United States). Ethanol (95%, v/v) was purchased from Merck Co. (Santa Ana, CA, United States). Water was double distilled and deionized. Human cervical carcinoma (HeLa) cells were provided by the Central Laboratory at the affiliated Xiangyang No.1 People’s Hospital of Hubei University of Medicine (Shiyan, China). HeLa cells were grown in 175 cm^2^ glass culture flasks dissolved in 30 ml of DMEM supplemented with 10% (v/v) fetal bovine serum (FBS) and 100 IU/ ml penicillin G sodium and 100 mg/ml streptomycin sulfate. The cells were stored in an incubator at 37°C with 5% CO_2_/95% air humidified atmosphere, subcultured three times per week, and used for the experiments when in exponential growth phase. Rabbit anti-human HPV18 E6, mouse anti-human P53, mouse anti-human Rb1, rabbit anti-human β-actin, and mouse anti-human GAPDH antibodies were purchased from Absin Biotechnology Co., Ltd (Shanghai, China). Rabbit anti-human HPV18 E7 antibody was purchased from Abcam (Cambridge, United Kingdom).

### Preparation of RES-HP-β-CD Complexes and Physical Mixtures

An inclusion complex of 1:1 M ratio was prepared by co-evaporation. Approximately 0.82 g of RES (Mw = 228 Da) and 5.54 g of HP-β-CD (Mw = 1540 Da) were dissolved in 40 ml of alcohol, respectively. RES solution and HP-β-CD solution were mixed in a sealed glass vial and agitated for 24 h at room temperature to obtain a clear solution. The sample was evaporated under reduced pressure in a rotary evaporator at 40°C to obtain a solid inclusion complex. A homogeneous physical mixture of RES and HP-β-CD (1:1 M ratio) was prepared by grinding the mixture with a mortar.

### Solubility Study

The solubility of RES and RHSD was studied based on a previously reported method. In brief, excess RES (20 mg) or RHSD complexes (140 mg) were mixed with water (5 ml) respectively, and the mixture was stirred at 25°C for 72 h. The samples were centrifuged at 15,000 ×*g* for 10 min to remove excess insoluble substance. The amount of RES was measured using HPLC.

### Particle Size Distribution

The particle size and polydispersity indexes (PDI) of HP-β-CD and RHSD were measured by Nano ZS90 laser light scattering instrument (Malvern, PA, United States). Briefly, 0.8 mg/ml of HP-β-CD and RHSD aqueous solutions were prepared, respectively. After filtered through a 0.45 um hydrophilic membrane filter, the samples were examined.

### Dissolution Study

The dissolution experiments were performed in normal saline by rotating basket method. Briefly, in each dissolution test, RES (25 mg) and RHSD (175 mg) were weighed and added into dissolution medium in a cell of dissolution tester (Rcz-6c3, Shanghai Huanghai Pharmaceutical Inspection Instrument Co., Ltd: Shanghai, China) and stirred with a speed of 100 rpm at 37°C. Aliquots (5 ml each) were withdrawn at 5, 10, 15, 20, 30, 45, 60, 90 and 120 min and immediately filtrated through a membrane filter (0.45 um). At each sampling time, an equal volume of fresh medium was added to the tester cell and the correction for the cumulative dilution was calculated. The concentrations of RES in the filtrated solutions were measured by UV-spectrophotometer at 306 um.

### Characterization of Inclusion Complex

#### Fourier Transform Infrared Spectroscopy

RES, HP-β-CD, physical mixture, and RHSD complex were measured with an FT-IR spectrometer (Thermo Fisher Scientific, Nicolet iS10, United States). The scanning scope was in the range of 4000–400 cm^−1^. All samples were applied to the spectrometer directly.

#### Powder X-Ray Diffraction

The PXRD spectra of RES, HP-β-CD, physical mixture, and RHSD were recorded on an X-ray diffractometer (Bruker) with Cu Kα radiation. The voltage and current were 45 kV and 40 mA, respectively. The scan rate was 16°/min between 3° and 60° in the 2θ angle range.

#### Differential Scanning Calorimetry

RES, HP-β-CD, physical mixture, and RHSD were accurately weighed separately. Their DSC curves were measured with a Q200 differential scanning calorimeter (TA, United States) at 10°C/min between 30 and 320°C in nitrogen atmosphere. An empty pan was used as the reference for the test.

#### ^1^H Nuclear Magnetic Resonance

The ^1^H NMR spectra of Res, HP-β-CD and RHSD complex spectra were tested with a Bruker Avance 600 spectrometer (Germany) at 600 MHz all samples were dissolved with DMSO-*d*_6_.

### Anti-tumor Efficacy of RES/HP-β-CD *In Vivo*

**FIGURE 1:**
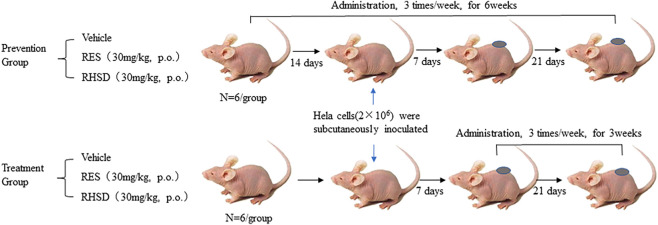
Schematic representation of BALB/C female nude mice of cervical cancer model derived from tumor HeLa cells *in vivo* and subsequent treatment cycle.

Female athymic BALB/C nude mice weighing 14–20 g (4–6 weeks) were purchased from Hunan SJA Laboratory Animal Co., Ltd (Changsha, China). Mice were housed at a controlled temperature of 20–22°C, with 50–60% relative humidity, and fed with a standard laboratory chow and tap water ad libitum. Mouse xenograft experiments in this study were complied with the ARRIVE guidelines and were conducted in accordance with the U.K. Animals (Scientific Procedures) Act, 1986 and associated guidelines. This study was approved by the Ethical Committee for Animal Experimentation of Xiangyang No.1 People’s Hospital (NO. 2017DW008). Athymic BALB/C were divided into the prevention group and treatment group (18 animals each group, Figure 1). Every group involved the mice being randomly divided into three subgroups: vehicle (normal saline containing 1% ethanol), RES (30 mg/ kg, p. o.), and RHSD (30 mg/ kg, p. o.). Each subgroup comprised six mice. The HeLa cell suspension containing 2 × 10^6^ cells in sterile saline was subcutaneously injected in the right flank of athymic BALB/C. Administration cycles were initiated in the treatment group when the tumor size reached approximately 100 mm^3^ (in 7 days). After 3 weeks, the mice were sacrificed. In the prevention group, the administration cycles were 42 days, and tumors were implanted on the 15th day after administration. The mice were sacrificed 4 weeks later.

Animals’ tumor volumes and body weight were measured every 3 days by using the formula as previously described (Ao et al., 2019): tumor volume = (length) × (width)^2^ × 0.5. The tumor-bearing mice were examined every 3 days for tumor growth by using a caliper. At the end of the administration, all mice were sacrificed by cervical dislocation, and tumors were isolated and weighed. RNA and protein were extracted and fixed in paraformaldehyde. Each tumor nodule was confirmed by hematoxylin–eosin staining.

### Western Blot Analysis

Cancer tissues were homogenized in lysis buffer composed of 10 mM Tris-HCl (pH 7.5), 150 mM NaCl, 1 mM EDTA, 1 mM EDTA, 50 mM NaF, 0.5 mM phenylmethylsulfonylfluoride, 1 mM sodium vanadate, 1% Triton X-100, 0.5% Nonidet P-40, and 1 μg/ml aprotinin. Samples were centrifuged at 12,000 ×*g* for 15 min at 4°C. An aliquot of the supernatant was used to determine protein concentration (Bio-Rad DC Protein Assay, Bio-Rad Laboratories, Hercules, CA, United States). Protein aliquots were mixed with 5× loading buffer, electrophoresed on SDS-polyacrylamide gels, and transferred electrophoretically onto polyvinylidene difluoride membranes. The membranes were incubated with HPV18 E6, HPV18 E7, P53, Rb1, β-actin, and GAPDH antibodies for overnight at 4°C. The blots were washed with tris-buffered saline and Tween-20, incubated with corresponding horseradish peroxidase-conjugated secondary antibody (Santa Cruz Biotechnology, Santa Cruz, CA, United States) for 1 h at room temperature, and developed using the ECL substrate (Thermo Fisher Scientific, Waltham, MA, United States). The relative amount of protein on the blots was determined by densitometry by Lab Works software (UVP, Upland, CA, United States). GAPDH was used as a loading control. The relative expression of HPV18 E6, HPV18 E7, P53, and Rb1 was computed as follows: sample gray value/GAPDH gray value. Primary antibodies were used at 1:500–1000 dilution, and secondary antibodies were used at 1:10,000 dilution.

### Real-Time Polymerase Chain Reaction

Total RNA was isolated from cancer tissues using TRIzol reagent (Invitrogen). Reverse transcription was performed with 2 μg of total RNA and M-MLV reverse transcriptase (Promega, Madi-son, WI, United States). PCR amplification was performed using Taq polymerase (Bio-Rad, Hercules, CA, United States) under the following conditions: denaturation at 94°C for 5 min, followed by 40 cycles of denaturation for 30 s at 94°C and annealing and extension for 60 s at 60°C. Real-time PCR was performed using the SYBR Green PCR Master Mix (Bio-Rad, Hercules, CA, United States) and a real-time PCR apparatus (IBA 7500, United States). The HPV18 E6 gene was amplified using the primers 5′-GCC​AGA​AAC​CGT​TGA​ATC​C-3' (forward) and 5′-AGT​CTT​TCC​TGT​CGT​GCT​CG-3′ (reverse), whereas the HPV18 E7 gene was amplified using the primers 5′-GCA​TGG​ACC​TAA​GGC​AAC​A-3′ (forward) and 5′-CTC​GTC​GGG​CTG​GTA​AAT-3′ (reverse). GAPDH was used as an internal marker and amplified using the primers 5′-AGA​AGG​CTG​GGG​CTC​ATT​TG-3′ (forward) and 5′-AGG​GGC​CAT​C-CAC​AGT​CTT​C-3′ (reverse). To calculate differential gene expression, we used the 2^−△△Ct^ formula.

### H&E Staining and IHC Assay

Live and kidney organs obtained by 4% paraformaldehyde fixation were processed by paraffin section. The sections were stained with H&E and analyzed by histology and immunohistochemistry to evaluate organ metastasis, and examined under an inverted microscope (OLYPUS 75X Microscope, Tokyo, Japan). For the IHC assay of the expression levels of HPV18 E6, HPV18 E7, P53, and Rb1, the tumor paraffin-embedded sections were incubated with antihuman HPV18 E6, HPV18 E7, P53, and Rb1 primary antibodies. A biotinylated goat anti-rabbit antibody and rabbit anti-mouse antibody were used as secondary antibodies. The slides were washed with PBS and incubated with diaminobenzidine chromogen for 3–5 min to yield a dark brown color. The sections were counter-stained with hematoxylin for microscopic observation (OLYPUS 75X Microscope, Tokyo, Japan). The expression levels of HPV18 E6, HPV18 E7, P53, and Rb1 were calculated in five randomly selected areas in each tumor sample as the number of positive cells/total were counted at ×400 magnification. Cells with moderate and strong brownish cytoplasmic staining were considered positive, whereas cells with unstained or weakly stained cytoplasm were considered negative.

### Statistical Analysis

All the results are expressed as the mean ± standard deviation (SD) for at least three independent experiments. Statistical differences were analyzed by independent sample t-tests. The differences between the experimental and control groups were compared by one-way ANOVA, followed by Dunnett’s multiple comparisons test. Stata 7.0 (Stata Corp LP, College Station, TX, United States) was used. Values of *P* < 0.05 were considered statistically significant.

## Results

### Solubilization and Dissolution Studies

**FIGURE 2:**
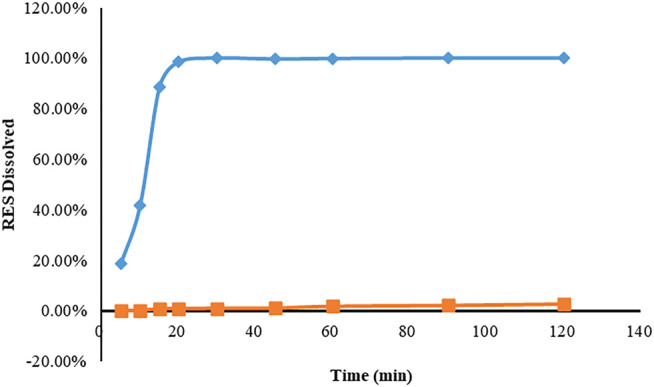
Dissolution profiles of resveratrol (■) and inclusion complex (◆) in normal saline after 120 min. The results were expressed according to the mean and standard deviation (n = 3) of the analyzes.

The solubility of RHSD was 4.18 ± 0.44 mg in pure water at 25°C, which was 438.6 times higher than that of free RES (0.00953 ± 0.004 mg), indicating that HP-β-CD was a more efficient solubilizer than raw RES. In Figure 2, the dissolution profile of the RHSD appeared to be much better than that of the RES. About 100% of the RES of complex amount dissolved within 10–20 min, while Less than 3% free RES was dissolved within 120 min.

### Characterization of Inclusion Complex

**FIGURE 3:**
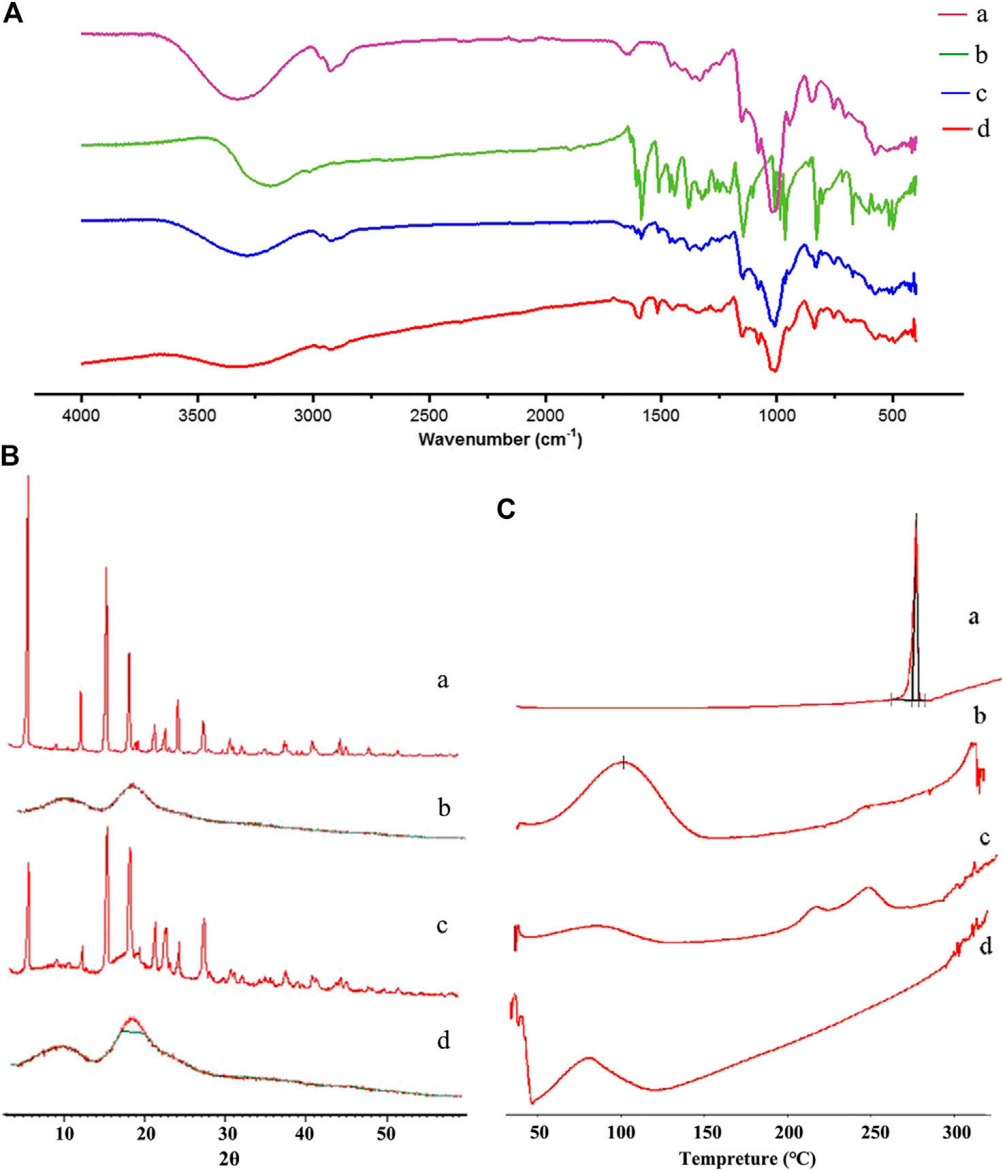
FT-IR **(A)**, PXRD **(B)**, and DSC **(C)** spectra of RES **(A)**, HP-β-CD **(B)**, physical mixture **(C)**, and RHSD inclusion complex **(D)**.

IR Figure 3A displays the IR spectra of free RES, HP-β-CD, physical mixture, and RHSD complex. Variations in IR peaks of guest or host are strong evidence for the occurrence of the complex. The IR characteristic band of O-H at 3185 cm^−1^ and the peaks at 1605, 1583, and 1510 cm^−1^ indicated the benzene ring of raw RES; these values were similar to previously reported findings (Zhang et al., 2013). The IR spectra of the physical mixture (Figure 3A-c) was the sum of the spectrum of HP-β-CD and RES, showing no or a minor interaction in the physical mixture. The IR spectrum of the inclusion complex (Figure 3A-d) supported the formation of the complex. No absorption peaks were found at 1605 cm^−1^. The peak at 1583 cm^−1^ shifted to 1593 cm^−1^, and that at 1510 cm^−1^ shifted to 1515 cm^−1^. The disappearance and shift characteristics of the spectrum revealed the formation of the RHSD inclusion complex.

PXRD Figure 3B displays the PXRD spectra of free RES, HP-β-CD, physical mixture, and RHSD complex. In the RES diffractogram, the diffraction peaks of Figures 3B-a indicated the crystalline form of RES. No crystalline peak was found in the HP-β-CD diffractogram (Figures 3B-b), indicating the amorphous form of HP-β-CD. The physical mixture showed the sum diffraction peaks of two free components (Figures 3B-c). RHSD did not show a diffraction peak of RES (Figures 3B-d), indicating new amorphous formation.

DSC Figure 3C displays the DSC curves of free RES, HP-β-CD, physical mixture, and RHSD. In the RES curve, the sharp endothermic peak at 264°C, which was the melting point of RES, showed its crystalline form (Figures 3C). The thermogram of the HP-β-CD curve presented a wide peak at 108°C (Figures 3C-b), which suggested a release of water molecules. The physical mixture displayed the sum thermogram peaks of two free components, which indicated that free RES and HP-β-CD did not interact with each other (Figures 3C-c). In the RHSD complex, the amorphous state was confirmed by the disappearance of the characteristic RES peak (Figures 3C-d). All the above findings supported the formation of stable complexes between RES and HP-β-CD.

**FIGURE 4:**
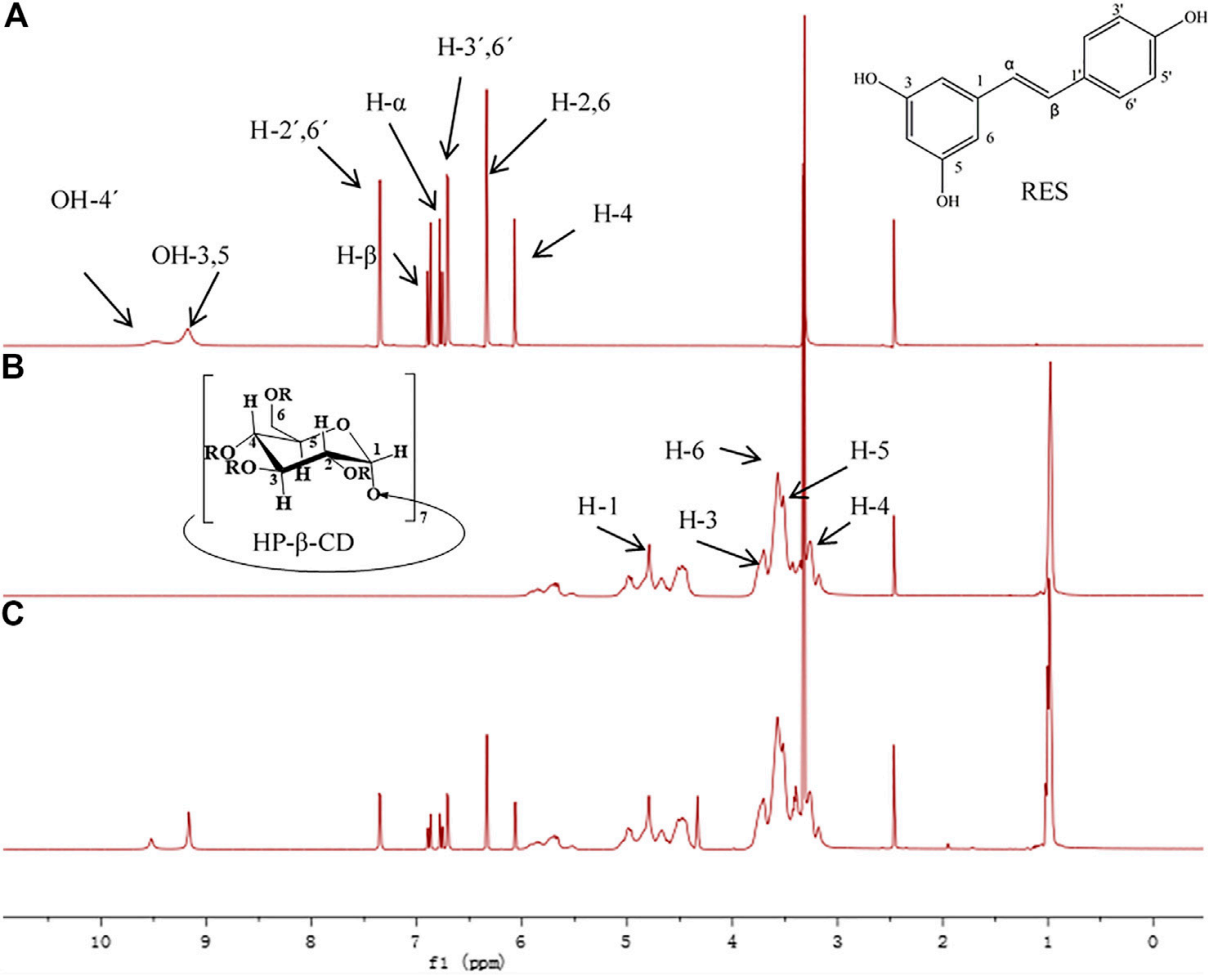
The ^1^H NMR spectra of resveratrol **(A)**, HP-β-CD **(B)** and the RHSD inclusion complex **(C)**.

^1^H NMR Figure 4 displays sectra of free Res, HP-β-CD and RHSD complex. All chemical shifts and variation of the complexes are listed in Table 1. The chemical shifts of H-2, H-6 and H-4 of Res have little variation by ∆δ -0.005 and -0.004 ppm respectively after complexation with HP-β-CD. These upfield shifts show that the benzene ring of Res enter the CD cavities. With the guest molecule into CD cavity, inside protons (H-3, H-5) have upfield shift by ∆δ -0.003 ppm and -0.002 ppm for electron cloud shielding effection, while outside protons (H-1, H-2 and H-4) have no shift upon complexation. The dates from ^1^H NMR indicate that Res is embedded in to the HP-β-CD cavity.

**TABLE 1:** Variation of the ^1^H NMR chemical shifts (ppm) of Res and HP-β-CD protons in free and complex states determined in DMSO-d6.

Substance	Proton	Free (δ)	Complex (δ)	∆δ
Res	H-2,6	6.338	6.333	−0.005
	H-4	6.069	6.065	−0.004
	α	6.785	6.784	−0.001
	β	6.898	6.897	−0.001
	H-2′, 6′	7.358	7.358	0
	H-3′, 5′	6.716	6.715	+0.001
HP-β-CD	H-1	4.791	4.791	0
	H-2	3.396	3.396	0
	H-3	3.705	3.702	−0.003
	H-4	3.259	3.259	0
	H-5	3.517	3.515	−0.002
	H-6	3.570	3.570	0

### Particle Size Distribution Analysis

Cyclodextrins and their complexes can form large water-soluble aggregates to solubilize lipophilic water-insoluble drugs in aqueous solutions. The values of diameter and PDI of cyclodextrins are different after forming complexes. The value of PDI close to zero (<0.10) indicates little variability in size (monodisperse), whereas values greater than 0.10 indicate polydisperse systems (Li et al., 2015). As shown in Table 2. The differences values of PDI, and the higher diameters of complexes than HP-β-CD suggested RES was embedded into HP-β-CD. Variability in PDI could be due to the tendency of the inclusion complex particles to agglomerate, because of the lack of significant net charge (no repulsive forces) to prevent particle agglomeration. Which maybe result the larger particle diameter of RHSD than HP-β-CD.

**TABLE 2:** Polydispersity indexes (PDI) and average diameter of HP-β-CD and the RHSD complex.

Sample	Polydispersity index	Particle diameter (nm)
HP-β-CD	0.615 ± 0.334	119.1 ± 22.370
RHSD	0.609 ± 0.176	227.2 ± 6.568

### RES/HP-β-CD and RES Inhibited Cervical Tumor Growth in the Mice Model

**FIGURE 5:**
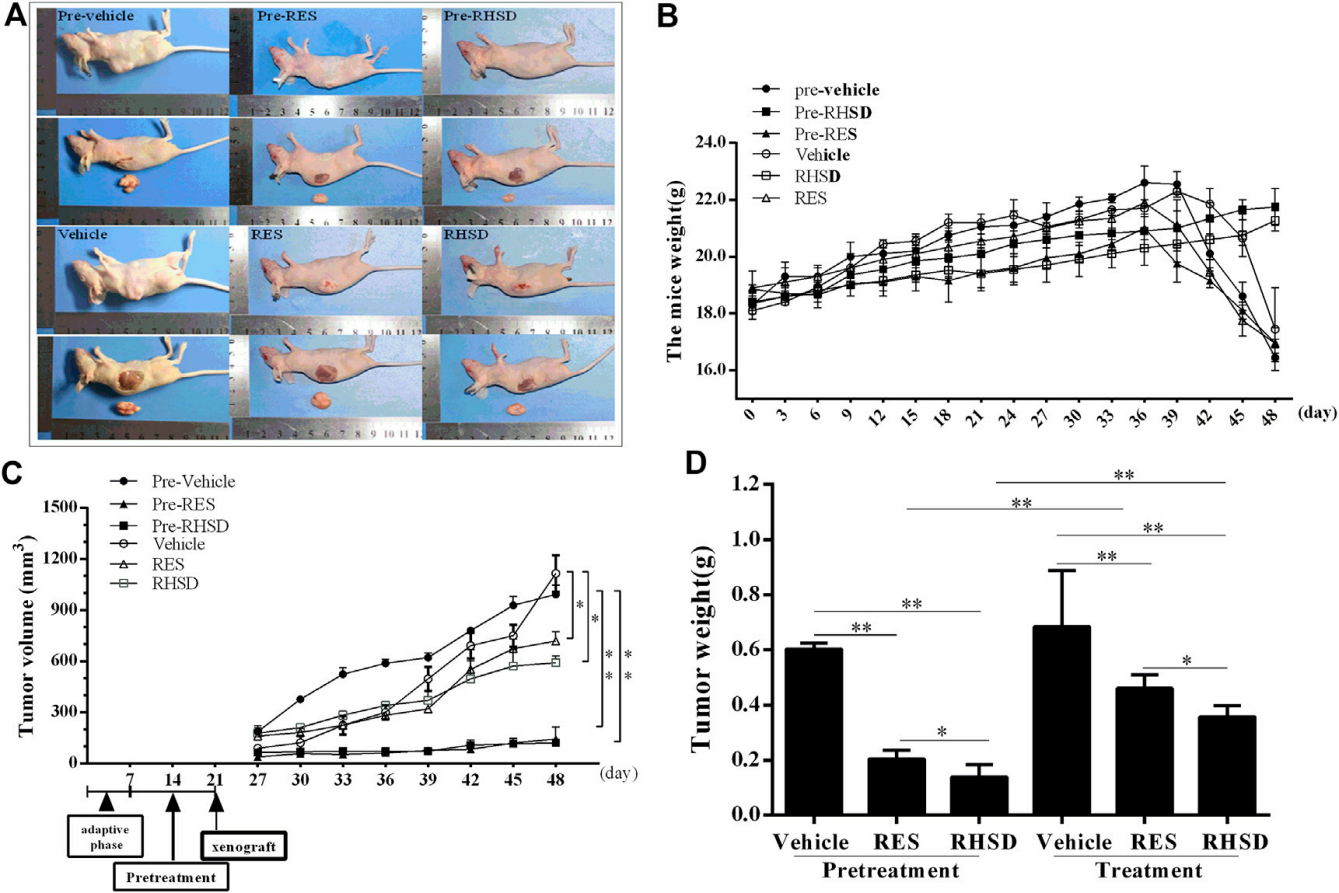
Effects of RES and RSHD on cervical cancer tumor growth *in vivo* **(A)** Typical image of subcutaneous tumor formation in nude mice **(B)** Mice weight **(C)** Tumor volume **(D)** Tumor weight. The data were presented as the mean ± S.D. *n* = 6/group, *p < 0.05, **p < 0.01.

Compared with free RES, the treatment and prevention efficacy of RHSD on HeLa xenografts in mice were evaluated. The tumor volume, tumor weight, and mice weight were measured and plotted after each treatment. As shown in Figure 5C, tumor growth in the prevention group was considerably slower than that in the treatment and vehicle groups. At the last day, the tumor volumes of the pre-RHSD and pre-RES groups were 130.23 ± 38.12 (*p* < 0.01) and 353.31 ± 43.52 mm^3^ (*p* < 0.01), respectively. Meanwhile, the tumor volumes of the RHSD and RES treatment groups were 591.61 ± 112.78 (*p* < 0.05) and 719.52 ± 90.58 mm^3^ (*p* < 0.05), respectively, whereas those of the pre-vehicle and vehicle groups were 992.3 ± 73.16 and 1102 ± 129.37 mm^3^, respectively. The changes in tumor weight (Figure 5D) were the same as those in tumor volume. The tumor weights of the pre-RHSD and pre-RES groups were 0.137 ± 0.077 (*p* < 0.01) and 0.203 ± 0.054 g (*p* < 0.01), respectively. Meanwhile, the tumor weights of the RHSD and RES treatment groups were 0.357 ± 0.070 (*p* < 0.01) and 0.459 ± 0.096 g (*p* < 0.01), respectively. The tumor weights of the pre-vehicle and vehicle groups were 0.541 ± 0.073 and 0.674 ± 0.186 g, respectively. The tumor weight of the pre-RHSD group was lighter than that of the RHSD group (*p* < 0.01). The same trend was observed in the pre-RES and RES groups. In addition, the tumor weight of RHSD was lighter than that of RES in the prevention and treatment groups (*p* < 0.05). The mice maintained a slow rate of weight gain for the two groups in the early stage of the experiment (36 days; Figure 5B), and the pre-RES, RES, and vehicle groups demonstrated a sharp drop. However, the weights of the pre-RHSD and RES groups maintained an upward trend. At the last day, the mice weights of the pre-RHSD and RHSD groups were 21.75 ± 1.16 and 21.27 ± 0.93 g, respectively, whereas those of the pre-RES and RES groups were 16.9 ± 1.01 and 16.95 ± 1.35 g, respectively. The mice weight of the vehicle group was 16.47 ± 1.66 g. The average mice weight between each group did not significantly differ. On the basis of the results, the effects of RHSD on the tumor volume and weight were stronger than the effects of RES in the prevention and treatment groups compared with the vehicle group. Therefore, the RHSD complex enhanced prevention and antitumor activity compared with free RES.

### RES/HP-β-CD and RES Inhibited the Expression of HPV18 E6/E7 and Increased the Expression of P53 and Rb1 *in vivo*

**FIGURE 6:**
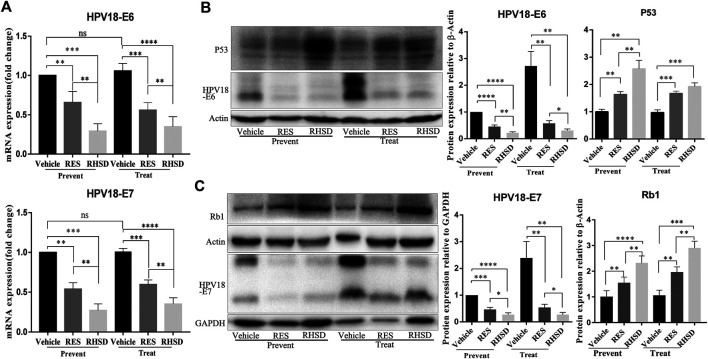
Expression of HPV18 E6, E7, P53, and Rb1 in cervical cancer tissues of mice model after RES or RHSD treatment **(A)** HPV18 E6 and E7 mRNA levels in cancer tissues were determined using real-time RT-PCR **(B)** HPV18 E6 and P53 protein levels in cancer tissues were determined by Western blot. The histogram on the right is the quantitative analysis of the results **(C)** HPV18 E7 and Rb1 protein levels in cancer tissues were determined by Western blot. The histogram on the right is the quantitative analysis of the results. The data were presented as the mean ± S.D. for three different experiments performed in triplicate. **p < 0.01, ***p < 0.001, and ****p < 0.0001.

**FIGURE 7:**
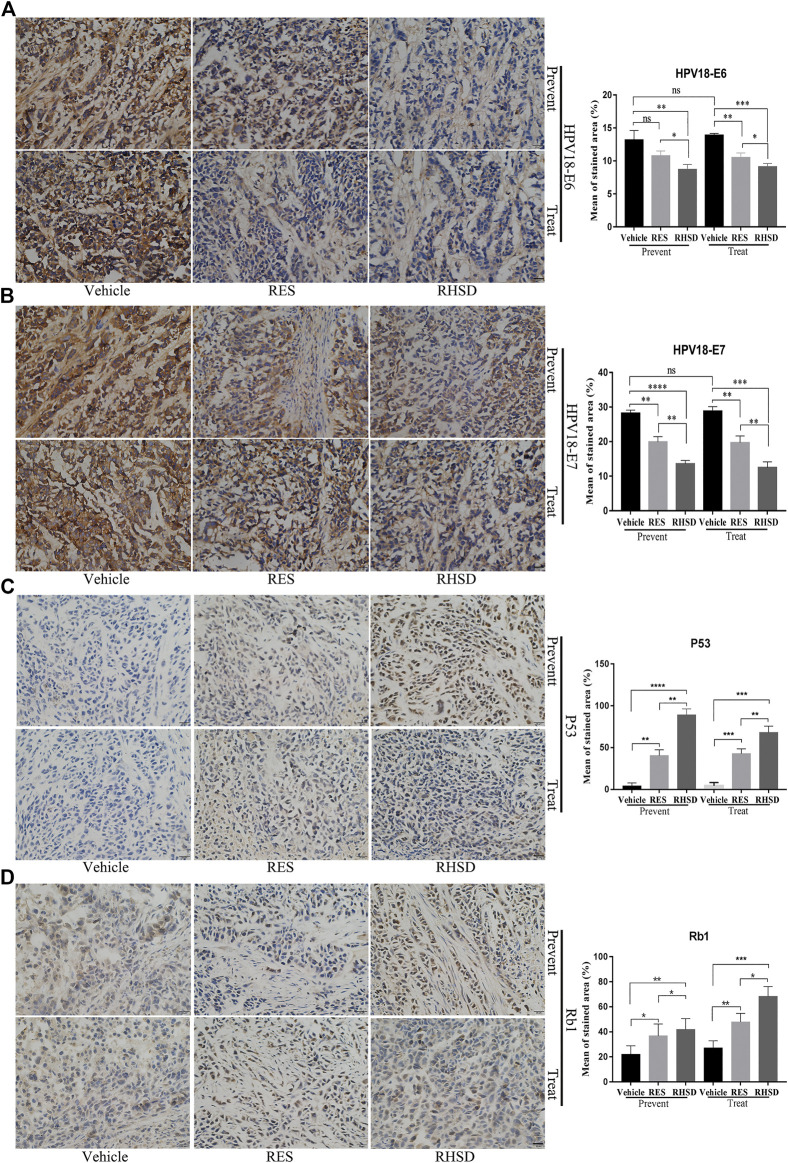
Distribution and expression of HPV18 E6, E7, P53, and Rb1 in cancer tissues. IHC staining of HPV18 E6, E7, P53, and Rb1 protein in tumor samples monoclonal antibody. Tumor sections immunostained with E6 **(A)**, E7 **(B)**, P53 **(C)**, and Rb1 **(D)** antibodies in mice treated with RES or RHSD (scale bar = 20 μm). The histogram on the right is the quantitative analysis of the results. The data were presented as the mean ± S.D. for three different experiments performed in triplicate. *p < 0.05, **p < 0.01, ***p < 0.001, ****p < 0.0001.

E6 and E7 proteins have been reported to play a critical role in the proliferation of cervical cancer cells. The suppression of viral E6 and E7 oncogene expression has been linked to apoptosis in cervical cancer cells. These findings led us to investigate whether the E6 and E7 oncogenes are attenuated by RHSD and RES. As shown in Figure 6, the mRNA levels of E6 and E7 treated with RHSD were lower than those treated with RES (*p* < 0.01) compared with the vehicle (*p* < 0.001 and *p* < 0.01, respectively) in the prevention group (Figure 6A). These findings were similar to the mRNA levels of E6 and E7 in the treatment group (Figure 6A). Consistently, the Western blot results indicated that the protein levels of E6 were significantly decreased by RHSD compared with the vehicle in the prevention and treatment groups (*p* < 0.01 and *p* < 0.0001, respectively; Figure 6B). The levels of protein E7 were also significantly inhibited by RHSD compared with the vehicle group in the prevention and treatment groups (*p* < 0.01 and *p* < 0.0001, respectively). The levels of protein E7 were highly attenuated by RES compared with the vehicle group (*p* < 0.001 and *p* < 0.001, respectively; Figure 6C). Therefore, RHSD was more potent in downregulating E6 and E7 mRNA transcription and protein expression than RES. Tissue immunohistochemical tests showed consistent results (Figure 7). The E6 and E7 protein levels were very high in control tumor sections (Figures 7A,B), but the prevention and treatment groups showed much lower expression of E6 and E7. This result suggested that RHSD and RES could downregulate E6 and E7. The inhibition efficiency of RHSD on E6 and E7 was higher than that of RES (*p* < 0.01 and *p* < 0.01, respectively).

**FIGURE 8:**
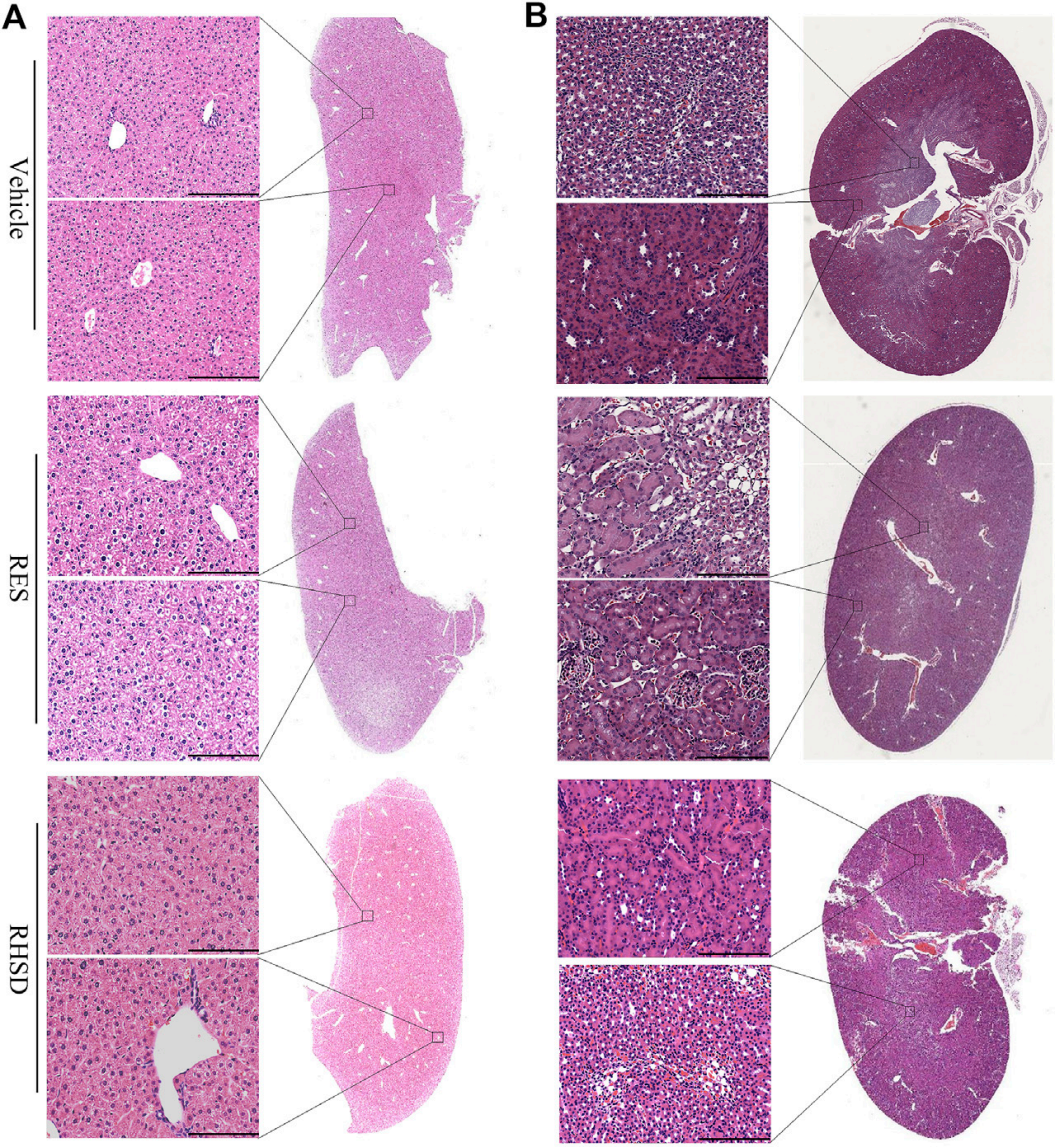
RES and RHSD are effective cervical cancer inhibitors with low toxicity. H&E staining of main organs of tumor-bearing nude mice after treatment. Full section scan and local magnification of Live **(A)** and kidney **(B)**, Scale bar: 75 μm.

P53 and Rb1 have been shown to trigger apoptosis in E6-and E7-harboring cervical cancer cells, respectively (Huh et al., 2007). The expression levels of P53 were improved by RHSD as compared with the vehicle in the prevention and treatment groups (*p* < 0.0001 and *p*< 0.01, respectively). P53 expression was also upregulated by RES compared with the vehicle (*p* < 0.01 and *p* < 0.001, respectively). The expression of P53 by RHSD was significantly higher than that by RES in the prevention group (*p* < 0.01; Figure 6B). The levels of Rb1 were strongly upregulated by RHSD than by RES (*p* < 0.01) in the prevention group compared with the vehicle (*p* < 0.0001 and *p* < 0.01, respectively). In the treatment group, the levels of Rb1 were also highly upregulated by RHSD than by RES (*p* < 0.01). In the treatment group, the expression of Rb1 was higher compared with the vehicle (*p* < 0.0001 and *p* < 0.001, respectively) by RHSD and RES, and Rb1 expression was upregulated by RHSD than by RES (*p* < 0.001; Figure 6C). The immunostaining results showed that tumors treated with RHSD had a significantly higher amount of activated P53 and Rb1 than tumors treated with RES and vehicle (Figures 7C,D). The amount of immunostaining cells indicated that *in vivo* RHSD and RES treatment appeared to activate the process of P53- and Rb1-mediated apoptosis, and the efficiency of RHSD to P53 and Rb1 was higher than that of RES (P <0.01 and P <0.05, respectively). These results demonstrated that treatment with RHSD possessed a more powerful ability on P53 and Rb1 than treatment with RES. Notably, the transcriptional inhibition of E6 and E7 by RES and RHSD promoted the recovery of P53 and Rb1 expression, respectively. In addition, H&E staining of live and kidney organs showed a normal appearance in treatment and control groups, without any significant signs of toxicity or inflammatory lesions, suggesting RHSD is as nontoxic as RES. (Figure 8).

## Discussion

In this study, we have elucidated for the first time the prevention and treatment of cervical cancer with the RHSD complex using the BALB/C female nude mice model of cervical cancer derived from HeLa cells. We demonstrated that the RHSD complex showed a higher efficacy in affecting tumor growth than RES alone. Notably, RHSD showed significant prevention effects compared with RES. Further analysis suggested that the RHSD complex significantly downregulated the HPV oncogenes E6 and E7 *in vivo* and upregulated the P53 and Rb1 levels. By comparison, RES showed less efficacy on these proteins than the RHSD complex.

RES, well-tolerated polyphenol with low toxicity to humans, is known to have proapoptotic, chemopreventive, and anticancer properties (Fulda, 2010). However, the low bioavailability of RES resulting from its poor water solubility limits its application. Our results showed that the solubility of RES increased (by more than 438.6-fold) significantly after the inclusion complex formed in a ratio of 1:1 with HP-β-CD, and the dissolution date of complex was also improved than free RES. The crystalline structure of RES was transformed into an amorphous form (proven by UV, IR, PXRD, DSC and ^1^H NMR).

Previous research demonstrated that RES was able to specifically target the essential oncoprotein E6 and induce cervical cancer cell death *in vitro*, and decreased the tumor size (83.5% compared to control) on TC1 murine model (Chatterjee et al., 2018; Chatterjee et al., 2019). But the efficacy of RHSD on cervical cancer has not been explored. To evaluate the treatment and prevention efficacy of the RHSD complex in an *in vivo* model of anti-cervical cancer, we established cervical tumors in nude mice and subjected them to RHSD inclusion complexes to evaluate the antitumor effects and mechanism of action compared with RES. At the end of the treatment cycle, RHSD decreased the tumor volume by 86.88% in the prevention group; this result was higher than that achieved by RES (64.39%) compared with the control. In the treatment group, the tumor volume was reduced by 40.38% by RHSD, and this percentage was higher than that by RES (27.4%). Thus, RHSD complexes demonstrated high efficacy in inhibiting the growth of HeLa cells *in vivo* compared with RES, and the effect of preventing tumor growth by RHSD was more prominent than that by RES. Our results implied that RES was effective in drastically shrinking a cervical cancer tumor model, which was in accordance with previous report (Chatterjee et al., 2019). In addition, the HP-β-CD enormously improved the pharmacological activity of RES *in vivo*, indicating the strong potential of RHSD to inhibit tumor growth in a mouse cervical cancer model. Therefore, complexes with HP-β-CD are a promising strategy for RES delivery systems.

The growth of HPV-positive cancer cells depends on the expression of oncoproteins. The E6/E7 oncoproteins are attractive therapeutic targets because of their rapid inhibitory effect on human papillomavirus-positive cancer cells. This cellular response is related to the regeneration of P53, which is an Rb1 anti-proliferative protein (Taghizadeh et al., 2019). The binding of E6 and P53 leads to the activation of P53, and the reactivation of P53 can inhibit cell proliferation and induce apoptosis in cervical cancer cells (Xiong et al., 2015). E7 is connected with the retinoblastoma (Rb1) protein; its inhibition leads to the upregulation of Rb1 and inhibition of tumorigenicity in CaSki cells (Choo et al., 2000). In the current study, the expression levels of the E6 and E7 oncogenes were analyzed by real-time PCR and Western blot in tissues. We found that RES and RHSD downregulated the mRNA of E6 and E7. The levels of E6 and E7 by RHSD were lower than those by RES, and this trend was also confirmed by Western blot. The difference between RHSD and RES in activating E6 and E7 inhibition was statistically signiffcant. Therefore, this study showed that E6 and E7 might play a crucial role in RHSD’s effects on the proliferation of cervical cancer cells. Interestingly, the expression of tumor suppressor proteins P53 and Rb1 were simultaneously increased by RHSD compared with the control group. These findings suggested that RHSD reduced tumor size by inhibiting the E6/P53 and E7/Rb1 pathways.

Given that paraffin blocks are valuable for future studies and review, immunohistochemistry for E6, E7, P53, and Rb1 protein expression was analyzed in this study. Our research revealed that E6 and E7 immunostaining were low in intensity by RHSD and RED compared with the vehicle group. The intensity of E6 and E7 staining was lower in the RHSD group than in the RES group. High intensity of P53 and Rb1 immunostaining was also found in the RHSD group than in the RES group. These results were consistent with the findings of qPCR and Western blot. These results further suggested that the restoration of P53 and Rb1 proteins induced by RHSD was dependent on E6 and E7. Moreover, HP-β-CD could improve the bioavailability of RES, and RHSD could effectively induce apoptosis mediated by the inhibition of E6/E7 expression.

## Conclusion

RES has a wide range of biological activity. Phase solubility studies demonstrated that HP-β-CD is an effective cyclodextrin for the development of an inclusion complex with RES. In *in vivo* study, we provide strong evidence that RHSD was effective in drastically shrinking a cervical cancer tumor model than free RES, which functioned through inhibition of the E6/P53 and E7/Rb pathways. Therefore, the RHSD inclusion complex has potential for development of a new oral administration drug for the treatment of cervical cancer.

## Data Availability

The datasets supporting the conclusions of this article are included within the article and its additional files.
